# Progress in the Regulation of Immune Cells in the Tumor Microenvironment by Bioactive Compounds of Traditional Chinese Medicine

**DOI:** 10.3390/molecules29102374

**Published:** 2024-05-17

**Authors:** Yuqian Chen, Wenshuang Fan, Yanyan Zhao, Meijun Liu, Linlin Hu, Weifen Zhang

**Affiliations:** 1 School of Pharmacy, Shandong Second Medical University, Weifang 261053, China; 15610605066@163.com (Y.C.); 13176220559@163.com (W.F.); yanyanzhao1999@163.com (Y.Z.); w15098523592@163.com (M.L.); 2 Shandong Engineering Research Center for Smart Materials and Regenerative Medicine, Weifang 261053, China

**Keywords:** Chinese medicine, bioactive compound, tumor microenvironment, immune cell, immune checkpoint, immunotherapy

## Abstract

The tumor microenvironment (TME) can aid tumor cells in evading surveillance and clearance by immune cells, creating an internal environment conducive to tumor cell growth. Consequently, there is a growing focus on researching anti-tumor immunity through the regulation of immune cells within the TME. Various bioactive compounds in traditional Chinese medicine (TCM) are known to alter the immune balance by modulating the activity of immune cells in the TME. In turn, this enhances the body’s immune response, thus promoting the effective elimination of tumor cells. This study aims to consolidate recent findings on the regulatory effects of bioactive compounds from TCM on immune cells within the TME. The bioactive compounds of TCM regulate the TME by modulating macrophages, dendritic cells, natural killer cells and T lymphocytes and their immune checkpoints. TCM has a long history of having been used in clinical practice in China. Chinese medicine contains various chemical constituents, including alkaloids, polysaccharides, saponins and flavonoids. These components activate various immune cells, thereby improving systemic functions and maintaining overall health. In this review, recent progress in relation to bioactive compounds derived from TCM will be covered, including TCM alkaloids, polysaccharides, saponins and flavonoids. This study provides a basis for further in-depth research and development in the field of anti-tumor immunomodulation using bioactive compounds from TCM.

## 1. Introduction

According to Global Cancer Statistics 2020, tumors have become the leading cause of human death, with 19.3 million new cases and nearly 10 million deaths worldwide in 2020 [1]. The development of tumors is closely linked to the immune function of the body. Immunotherapy, known for its remarkable effectiveness and minimal side effects, has emerged as the fourth anti-tumor therapy, following surgery, radiotherapy and chemotherapy [2]. In fact, it has been recognized as one of the top ten scientific breakthroughs before 2013 by the journal Science [3]. The efficacy of tumor immunotherapy is primarily influenced by the tumor microenvironment (TME), which refers to the complex and rich multi-cellular environment within tumor tissues. This environment includes immune cells, stromal cells, extracellular matrix and secreted factors, as well as the blood and lymphovascular networks. All these components collectively communicate with each other and with heterogeneous cancer cells [4]. The TME is characterized by acidity, lack of oxygen and accumulation of metabolites due to an incomplete vascular system and anaerobic fermentation. These conditions lead to immunosuppression, allowing tumor cells to evade immune surveillance and escape killing by immune cells [5]. Therefore, targeting immune cells in the tumor microenvironment is an effective approach in immunotherapy.

**Figure 1:**
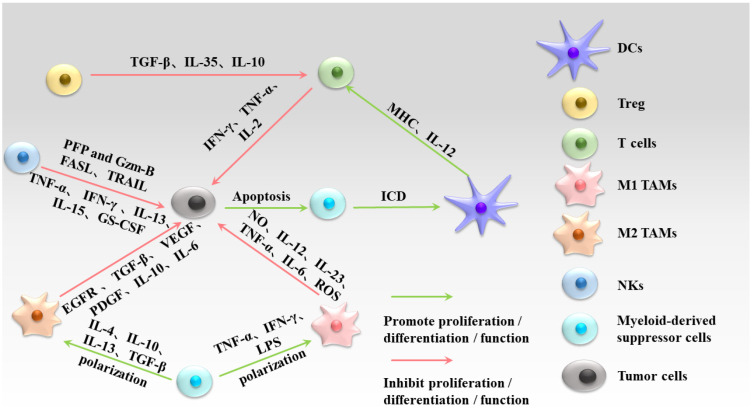
Tumor-associated immune cell interaction network. (DCs: dendritic cells; Treg: regulatory T cell; M1 TAMs: M1 tumor-associated macrophages; M2 TAMs: M2 tumor-associated macrophages; NKs: natural killer cells; TGF-β: transforming growth factor β; IL-35: interleukin 35; IL-10: interleukin 10; IFN-γ: interferon-γ; TNF-α: tumor necrosis factor α; IL-2: interleukin 12; MHC: major histocompatibility complex; ICD: immunogenic cell death; NO: nitric oxide; IL-23: interleukin 23; IL-6: interleukin 6; ROS: reactive oxygen species; LPS: lipopolysaccharide; IL-4; interleukin 4; IL-13: interleukin 13; EGFR: epidermal growth factor receptor; VEGF: vascular endothelial growth factor; PDGF: platelet-derived growth factor; PFP: perforin; Gzm-B: granzyme B).

Immune cells, such as tumor-associated macrophages (TAMs), dendritic cells (DCs), natural killer cells (NKs), T and B lymphocytes, neutrophils, and myeloid-derived suppressor cells, play a crucial role in immunotherapy (Figure 1) [6]. Unlike conventional chemotherapy, immunotherapy specifically targets and attacks tumor cells by utilizing immune cells both inside and outside the tumor microenvironment [7]. For instance, macrophages kill tumor cells by producing effector molecules and directly phagocytosing them, resulting in cytotoxicity [8]. Dendritic cells process and present antigens to T cells, activating them to induce apoptosis [9]. Natural killer cells directly recognize and eliminate tumor cells [6], while T cells secrete cytokines to induce apoptosis and directly kill tumor cells. Additionally, immune checkpoint blockade activates cytotoxic T cells and inhibits tumor cell proliferation [10]. These advancements have given rise to hopes of long-term survival in advanced malignant tumors such as lung and breast cancer and have significantly improved patient prognosis.

Traditional Chinese medicine (TCM) plays a significant role in Chinese medicine and has had a rich historical value in the Chinese nation’s fight against diseases over thousands of years. The bioactive compounds of Chinese medicines form the key factors of their therapeutic effects. Recent studies have revealed that various bioactive compounds of TCM possess anti-tumor immune properties by regulating immune cells within the tumor microenvironment. This discovery has deepened our understanding of the connection between traditional Chinese medicine and immunity [11]. However, further investigation is required to elucidate the mechanisms by which TCM’s bioactive compounds can reverse the immunosuppressive microenvironment and activate the immune response. By positively regulating immune cells or negatively regulating immunosuppressive cells, the bioactive compounds of traditional Chinese medicine can invert the immunosuppressive state of the tumor microenvironment and improve anti-tumor activity [12,13]. This emerging approach has opened new possibilities for tumor immunotherapy. This article focuses on immune cells in the tumor microenvironment and explores the regulatory effects of various bioactive compounds of traditional Chinese medicine, such as saponins, polysaccharides, diketones, terpenoids and quinones, on immune cells and immune checkpoints, including macrophages, dendritic cells, natural killer cells and T cells. The aim is to provide valuable insights for future research on immunotherapeutic applications of the bioactive compounds of traditional Chinese medicine.

## 2. Bioactive Compounds in Traditional Chinese Medicine

The bioactive compounds of TCM possess a wide range of biological activities, some of which, such as alkaloids, polysaccharides, saponins and flavonoids or their derivatives, have the potential to fight cancer alone or in combination with traditional anti-cancer drugs or therapies [14]. They have the potential to be excellent drugs for the prevention and treatment of cancer [15]. Alkaloids, some of the most important bioactive compounds in Chinese medicine, are a class of nitrogen-containing basic organic compounds, most of which have complex ring structures. At present, many existing clinical drugs, especially anti-cancer drugs, are derived from alkaloids and their derivatives [16]. Polysaccharides are carbohydrates that are involved in constituting life activities and maintaining biological functions. Polysaccharides are naturally occurring biomolecules consisting of more than 10 monosaccharides, and the monosaccharide composition, molecular weight and polysaccharide attachment affect their structure, which further influences their properties and functional mechanisms [17]. In recent years, a large number of studies have demonstrated that polysaccharides have a wide range of biological effects, including anti-viral, anti-aging, anti-coagulant, anti-tumor and immunostimulatory activities, among other biological effects [18]. Saponin compounds are widely distributed in terrestrial plants, and a small amount exist in marine organisms such as starfish and coral [19], which can be categorized into triterpenes and steroidal saponins according to their glycosidic carbon skeleton structures. Saponins are widely available in traditional Chinese medicine and have biological activities such as anti-tumor, anti-viral, anti-inflammatory, anti-bacterial, anti-pyretic and sedative activities [20]. Flavonoids are a class of polyphenolic compounds widely found in a variety of plants [21]. Flavonoids are plant-based compounds with antioxidant and anti-cancer potentials [22]. These bioactive compounds have been shown to modulate the TME in a variety of tumors (Table 1).

## 3. Regulation of Macrophages by the Bioactive Compounds in Traditional Chinese Medicine

Based on their phenotypes, macrophages are generally classified as M1 (classically activated) and M2 (alternatively activated) [50]. Classical M1 macrophage polarization is stimulated by tumor necrosis factor (TNF-α), interferon-gamma (IFN-γ) and bacterial lipopolysaccharide (LPS) [51]. Subsequently, M1 macrophages eliminate tumor cells by releasing NO, IL-12, IL-23, TNF-α, IL-6 and ROS [52]. On the other hand, M2-type macrophage polarization occurs in response to factors such as IL-4, IL-10, IL-13, TGF-β, glucocorticoids or immune complexes [53]. M2-type macrophages then secrete epidermal growth factor receptor (EGFR), transforming growth factor-β (TGF-β), vascular endothelial growth factor (VEGF), platelet-derived growth factor (PDGF), IL-10, IL-6 and arginase-1, promoting tumor angiogenesis and tumor progression [54]. In the tumor microenvironment, the majority of macrophages exhibit an immunosuppressive phenotype (M2), contributing to tumor proliferation, invasion and metastasis by inhibiting the tumor immune microenvironment, promoting stromal remodeling, and tumor angiogenesis, among other mechanisms. Therefore, modulating the immunosuppressive microenvironment by activating M1 macrophages and inhibiting M2 macrophages through the bioactive compounds of TCM can induce specific anti-tumor immunity and inhibit tumor metastasis [55,56,57].

Saponins and polysaccharides can regulate the phenotypes of tumor-associated macrophages (TAMs) by influencing signaling pathways such as TLR4/AMPK/NF-κB/STAT3 and directly modulating the expression of CD206 and VEGF (Table 2). Astragaloside IV, a natural saponin from *Astragalus membranaceus*, has been shown to inhibit M2 polarization through signaling pathways such as TLR4/NF-κB/STAT3, thereby regulating the tumor microenvironment and inhibiting Huh-7 nude mouse hepatocellular carcinoma tumor proliferation, invasion and migration in vivo [23]. Astragaloside IV (AS-IV) has also been reported to reduce Lewis lung cancer invasion, migration and angiogenesis in vivo by blocking M2 macrophage polarization through part of the AMPK signaling pathway [24]. Polyporus polysaccharide, a polysaccharide from *Polyporus umbellatus (Pers.) Fries.*, can enhance the bladder tumor microenvironment in vivo by regulating inflammatory cytokines and promoting the polarization of M1 macrophages [32,33,34]. Ginsenoside Rh2, a major saponin bioactive compound in ginseng, regulates TAM differentiation in the Lewis lung cancer microenvironment in vivo, promoting the shift of macrophages from M2 to M1 [25]. Ginseng-derived nanoparticles (GDNPs) isolated from ginseng can facilitate the transformation of Lewis lung cancer M1 TAMs in vivo. GDNPs promote the shift of M1 TAMs from “cold” to “hot”, synergizing with PD-1 monoclonal antibody immunotherapy for a greater effect [58]. Astragalus polysaccharide PG2, the bioactive compound polysaccharide in dried roots of *astragalus membranaceus,* enhances Lewis lung cancer M1 polarization in vivo and reduces IL-4-/IL-13-induced M2 polarization in a dose-dependent manner [35].

## 4. Regulation of Dendritic Cells by the Bioactive Compounds in Traditional Chinese Medicine

DCs serve as professional antigen-presenting cells, acting as the link between innate immunity and adaptive immunity. They play a pivotal role as initiators and regulators of immune responses. DCs possess a unique ability to transport tumor antigens to draining lymph nodes, initiating T cell activation, a crucial step for T cell-dependent immunity and immune checkpoint blockade (ICB) therapies [59]. Mature DCs secrete IL-12 and facilitate the differentiation of Th1 cells. Therefore, activating DCs in tumor patients is a promising avenue for promoting T cell activation and serves as an effective strategy for anti-tumor immunotherapy [60].

Studies have demonstrated that polysaccharides, terpenoids, flavonoids and quinones can promote DC maturation, enhance the antigen-presenting function of DCs and regulate the tumor immune microenvironment by directly influencing DC surface proteins, modulating the MyD88 pathway and inducing immunogenic cell death (ICD) [36,41,61,62,63,64,65] (Table 3). Highly purified mixed polysaccharide fractions from the roots of *Radix Astragalus* and *Radix Codonopsis* stimulate higher expression of CD80 and CD86 molecules on DCs, which is crucial for initial T-cell activation, leading to increased infiltration of CD8+ T cells in 4T1 breast cancer in vivo [36]. Gambogic acid nanoparticles improve the CT26 colorectal cancer immune microenvironment in vivo by stimulating DC maturation and increasing CD8+ T cell expression in the spleen [41]. Astragalus polysaccharide, a terpene from *Astragalus membranaceus,* can induce the activation of splenic DCs in vivo, further activating tumor-infiltrating T cells for anti-tumor effects [61]. Research by Liu et al. [62] has shown that cryptotanshinone (a terpene isolated from the TCM *Danshen/Salvia miltiorrhiza Bunge*) induces the maturation of Lewis lung cancer mouse DCs in a MyD88-dependent manner in vivo. *Cordyceps sinensis*, a medicinal fungus utilized in China for millennia, stimulates the expression of costimulatory molecules and pro-inflammatory cytokines in immature DCs in vitro, promoting their activation and maturation and enhancing T cell proliferation induced by DCs [63]. Plumbagin, a quinone extracted from the root of *Plumbago zeylanica L.*, induces ICD in HCC liver cancer in vivo by stimulating the maturation of DCs. Dihydrotanshinone I, a terpene from *Salvia miltiorrhiza* root, generates reactive oxygen species (ROS), enhancing plumbagin-mediated ICD activity and further promoting DC maturation [64]. Gambogic acid is a TCM flavonoid extracted from the *Garcinia hanburyi* tree that is known for its anti-tumor effects countering VEGF-mediated angiogenesis [65] and has been investigated for its role in the B16F10 melanoma and MC38 colon cancer immune microenvironments in vivo.

## 5. Regulation of Natural Killer Cells by the Bioactive Compounds in Traditional Chinese Medicine

NKs are cytotoxic lymphocytes with direct killing effects in the innate immune system. Similar in shape to T and B lymphocytes, they do not require pre-stimulation and can directly recognize and lyse tumor cells through surface receptors. Their anti-tumor effect does not require antigen sensitization, making them a potent tumor-killing cell alongside T cells and a crucial first line of defense in tumor immunity [66]. The anti-tumor mechanisms of NK cells include (1) releasing perforin (PFP) and granzyme B (Gzm-B) for target cell apoptosis; (2) regulating the death receptors FASL and TRAIL for apoptosis; and (3) secreting effector cytokines, such as IFN-γ, TNF-α, IL-5, IL-13 and G-CSF. NK cells play a pivotal role in tumor immune surveillance and can act as intermediary cells of adaptive immunity in tumor immunotherapy. However, if NK cells fail to monitor or eliminate tumor cells, this can easily lead to tumor immune escape.

Studies have demonstrated that the bioactive compounds of TCM, such as anthraquinones, plant-extracted glycoproteins and terpenes, can enhance tumor immune surveillance by inducing the secretion of perforin and granzyme B, boosting the proliferation ability and self-function of NKs [67] (Table 4). For instance, anthraquinone emodin has been found to enhance the killing effect of NKs on A549 lung cancer cells in vitro by influencing the balance of signals transmitted by NKs [68]. A glycoprotein isolated from Zanthoxylum piperitum DC (ZPDC) fruit induces tumor cells to secrete perforin and granzyme B, activating NKs and enhancing tumor immunosurveillance in liver cancer tissues in vivo [69]. Lupeol, a triterpene, has been shown to enhance the proliferative ability of NKs and their cytotoxicity against gastric cancer cells in vitro, possibly through up-regulating the expression of PFP, IFN-γ and CD107a [70]. The Wnt/β-catenin signaling pathway and the PI3K/Akt signaling pathway play an important role in promoting the proliferation of NKs.

## 6. Regulation of T-Cells by the Bioactive Compounds in Traditional Chinese Medicine

**Figure 2:**
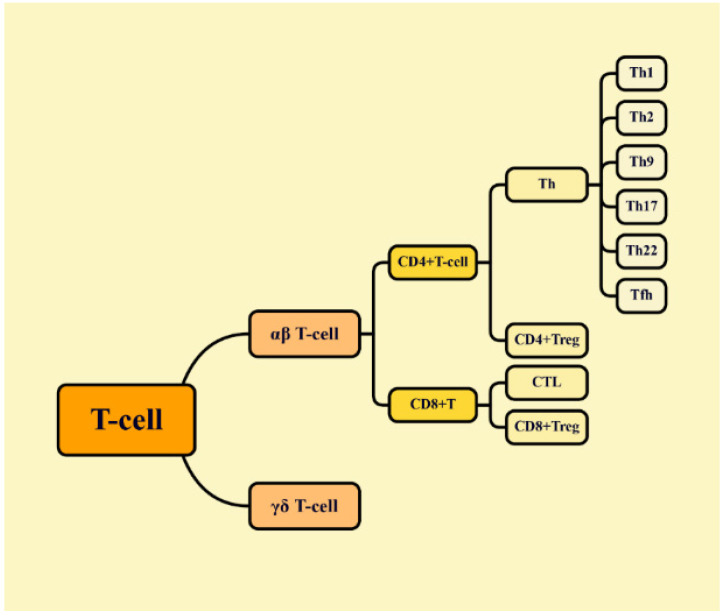
Classification of T cells. (CTL: cytotoxic T lymphocyte; Th: helper T cell; Tfh: follicular helper T cell; Treg: regulatory T cell).

As discussed earlier, T cells are classified into αβ T cells and γδ T cells (Figure 2). αβ T cells are further divided into CD4+ T cells (helper T cells) and CD8+ T cells (cytotoxic T cells (CTLs)), both of which play crucial roles in anti-tumor immunity. Initial CD4+ T cells mature through polarization by binding to compatibility complex II (MHC II) on the cell surface of dendritic cells (DCs), macrophages, B cells and other cells. Subsequently, they assist the anti-tumor process by recruiting CD8+ T cells, promoting their proliferation and enhancing T cell effector function through the production of IFN-γ-dependent chemokines and IL-2. CD4+ T cells can differentiate into a variety of effector T cells, including Th1, Th2, Th9, Th17, Th22, regulatory T cells (Tregs) and follicular helper T cells (Tfhs). Th1 cells primarily secrete cytokines such as TNF-α, IFN-γ and IL-2, playing a crucial role in anti-tumor immunity. Th2 cells produce IL-4, IL-5, IL-10, IL-13 and IL-31, assisting B lymphocytes in producing specific antibodies for humoral immunity. The balance of Th1/Th2 is pivotal in immune response regulation, with imbalances being associated with malignancies [71]. Regulatory T cells (Tregs) are a subset of CD4+ T cells that maintain immune homeostasis and impede tumor immune surveillance by producing the chemokines TGF-β, IL-35 and IL-10 [72,73]. Initial CD8+ T cells depend on MHC I presented by antigen-presenting cells to stimulate their maturation into CTLs, enabling them to directly kill tumor cells [74,75]. γδ T cells express TCRγ and δ chains and are involved in tumor progression. γδ T cells can kill tumor cells by releasing PFP and Gzm-B or through the T cell receptor-dependent pathway (antibody-dependent cytotoxicity) [76]. γδ T cells can interact with other immune cells by secreting pro-inflammatory cytokines such as TNF-α, IFN-γ, IL-17, IL-21 and IL-22 [77]. In summary, various types of T lymphocytes play crucial roles in tumor immunotherapy.

Previous studies have demonstrated that alkaloids, saponins, terpenoids and polysaccharides activate the tumor immune response, inhibiting tumorigenesis, progression and metastasis by enhancing the killing ability of γδ T cells and CTLs, regulating the ratio of Th1 and Th2 cells, and suppressing the activity of Treg cells [78] (Table 5). Ginseng extract saponins can modulate the immune system in various ways to achieve anti-tumor effects. For instance, Takei et al. [26] found that ginsenosides act on DCs, promoting the transformation of primitive T cells into Th1 cells, thereby increasing the production of a substantial amount of IFN-γ. Wu et al. [27] also reported that the levels of IFN-γ and IL-2 in the sera of H22 tumor-bearing mice treated with ginsenoside Rg3 were significantly higher than those in the tumor control group. Xia et al. [28] found that ginsenoside Rh2 (GRH2) exerted a significant anti-cancer effect on T-cell acute lymphoblastic leukemia cells by blocking the cell cycle, inhibiting cell growth, and promoting apoptosis and autophagy by targeting the PI3K/Akt/mTOR pathway. Saikosaponin A can transform Th2-type cells into Th1-type cells in breast cancer in rats [29], and Hemerocallis sinensis extract also significantly reduced the number of Treg cells and lowered the levels of IL-10 and TGF-β in a murine model of hepatocellular carcinoma. Sun et al. [37] reported that Ganoderma lucidum polysaccharides could inhibit mouse melanoma growth by inducing the differentiation of CTLs, triggering the production of granzyme B and perforin, and enhancing the cytotoxicity of CTLs to melanoma cells. Li et al. [38] found that Ganoderma lucidum polysaccharides significantly inhibited HCC liver cancer growth in mice through an increase in miR-125b expression, leading to an increase in the Notch1 signaling pathway and FoxP3 expression, thereby attenuating the inhibition of CTL proliferation by Treg cells. Licorice polysaccharide can inhibit H22 liver cancer proliferation by increasing the ratio of Th1 and Th2 cells through the activation of CD4+ and CD8+ T cells, decreasing the number of Treg cells, decreasing the expression of Foxp3, and increasing the Th1/Th2 ratio [39,40]. Wei et al. [47] reported that the TCM extract ligustrazine, an alkaloid, increased the expression of cytokines IFN-γ and IL-2 in lung cancer patients and reduced the expression of Th2 cytokines that mediate immune suppression. Elecampane (*Inula helenium L.*) enhances the anti-tumor activity of anti-PD-1 antibodies by increasing the proportion of CD8+ T cells and M1 macrophages in the MC38 colorectal cancer TME [79]. Scutellaria barbata extract can also significantly reduce the number of Treg cells and the levels of IL-10 and TGF-β in mice with liver cancer [80].

## 7. Regulation of Immune Checkpoints by Bioactive Compounds in Traditional Chinese Medicine

Immune checkpoints are a type of immunosuppressive molecule expressed on immune cells that regulate immune function to prevent autoimmunity [81]. During the tumor immune response, tumor cells can exploit immune checkpoints to inhibit T cell-mediated immune attacks [82]. CTLA-4, PD-L1 and others are checkpoint proteins that hinder T cell immunity, regulating immune recognition and escape. PD-1, present on the surface of αβ T cells and B cells, binds to PD-L1 and PD-L2 ligands on tumor cells, suppressing immune cell activation. By blocking PD-L1 on tumor cells and PD-1 on T cells, immunotherapy partially restores T cell function, mobilizing immune responses to identify and eliminate tumor cells, ultimately exerting anti-tumor effects [83,84,85]. CTLA-4 is another inhibitory receptor that inhibits T cell activation by binding to CD80/CD86 on antigen-presenting cells during costimulatory signaling. Coupled with ICB, TCM can improve the efficacy of inhibitors, reducing toxic and side effects and alleviating drug resistance in cancer treatment.

Studies have demonstrated that the bioactive compounds in TCM, such as saponins, terpenes, alkaloids and flavonoids, can down-regulate PD-L1 expression through various signaling pathways such as PI3K/Akt/mTOR, P38, NF-κB and JAK/STAT3, among others, thereby intervening in tumor immune escape (Table 6). These bioactive compounds activate immune cells to exert anti-tumor effects. For instance, Wang et al. [30] demonstrated that ginsenoside Rg3 (saponin) inhibits the expression of PD-L1 in LLC by suppressing the PI3K/Akt/mTOR pathway, blocking PD-L1-mediated immune escape, enhancing T cell immune response and inhibiting Lewis cell growth. Dong [42] found that hyperoside degrades c-Myc (a flavonoid), down-regulating the expression of immune checkpoints PD-L1 and CD47, in addition to reshaping the MC38 colorectal cancer immune microenvironment in vivo to induce anti-colorectal cancer effects. Qu et al. [31] reported that Astragaloside IV (saponin) inhibits PD-1 and PD-L1 expression through the P38 signaling pathway, reducing invasion and migration of cervical cancer HeLa cells. Peng et al. [43] found that (-)-Sativan (SA), a flavonoid from *Spatholobus suberectus Dunn.*, inhibits PD-L1 expression by up-regulating miR-200c, thereby inhibiting breast cancer tumor growth in vitro. Jing et al. [44] found that quercetin dihydrate attenuates the effect of PD-L1 on T cells by inhibiting PD-1/PD-L1 interaction. Li et al. [45] further demonstrated that quercetin reduces PD-L1 in tumor cells by inhibiting the JAK2/STAT3 pathway, thereby enhancing CTL activity in Lewis lung cancer mice. Jiang et al. [46] demonstrated that the flavonoids apigenin and lignans, which are abundant in fruits and vegetables, significantly inhibit the proliferation of lung cancer in KRAS mutants and down-regulate IFN-γ-induced PD-L1 expression. Liu et al. [48] found that the alkaloid berberine (BBR) degrades PD-L1 and activates tumor-infiltrating T-cell activation in Lewis lung cancer. The novel alkaloid Evodiamine, isolated from the fruit of the Tetradium tree, inhibits non-small cell lung cancer by increasing CD8+ T cell activity and down-regulating MUC1-C/PD-L1 [49]. Wan et al. [86] discovered that paeoniflorin (terpenoid) inhibits PD-L1 expression through the JAK/STAT3 signaling pathway, significantly reducing IFN-γ-induced PD-L1 up-regulation in HepG2 cells. Qiu et al. [87] found that the terpenoid Celastrol induces ICDs, activates DCs, recruits cytotoxic CD8+ T cells and NKs, and down-regulates PD-L1 expression through the NF-κB pathway for effective systemic immunotherapy in B16F10 melanoma mice. Andrographolide (a terpenoid) inhibits NSCLC tumor growth by inhibiting the phosphorylation of STAT3 and p62 accumulation, regulating selective autophagic degradation of PD-L1, and increasing the infiltration and function of CD8+ T cells [88]. Yu et al. [89] explored the effects of Caesalpinia sappan and Radix Astragali, two medicinal herbs commonly used in TCM, on CD4+CD25+ Treg cells and related regulatory molecules in a Lewis lung cancer mouse model. The authors found that TCM intervention significantly inhibits tumor growth and metastasis, possibly by inhibiting CTLA-4 in tumor tissues, reducing Treg cell numbers and function, improving immune tolerance, and inhibiting tumor growth and metastasis.

## 8. Conclusions and Perspective

TCM has a long history of clinical application in China. The medicinal herbs used in TCM contain a diverse array of chemical components, such as alkaloids, polysaccharides, glycosides and flavonoids, among others. These components activate various immune cells (TAMs, DCs, NKs, T cells, etc.), thereby enhancing systemic functions to maintain overall health. The bioactive compounds in TCM exert bidirectional regulation of immune cells within the TME, proving their use to be an effective approach in tumor immunotherapy. Positive regulation includes inducing M1 macrophage polarization, promoting DC differentiation and maturation, activating NK cells, encouraging Th1 transformation of primitive T cells, and inducing CTL differentiation. Conversely, negative regulation involves inhibiting M2 macrophage polarization, increasing the Th1/Th2 ratio, suppressing Treg function, hindering Th2 transformation of primitive T cells and impeding immune checkpoint activation in T cells. These regulatory effects are not limited to a single receptor or a single signaling pathway, but also include multi-level, multi-target and multi-pathway regulation of immune cells. A growing body of evidence has recently highlighted the advantages of using TCM to improve the body’s immune function, which has important clinical significance for patients with advanced tumors whose immune system functions are significantly compromised [90]. However, due to the complexity of TCM-based immunotherapy, coupled with an immature understanding of its underlying mechanisms and usage strategies, additional studies must be conducted before these novel therapies can be safely applied in a clinical setting. To advance research on immune regulation by TCM, the following steps should be taken:

(1) Construction of a scientific purification system for the bioactive compounds of TCM. The bioactive compounds of TCM are not only relatively complex but are also often difficult to detect and characterize due to their low contents. Modern analytical methods can be used to conduct a detailed analysis of the components of TCM (bioactive compounds, toxic components, etc.), accurately and comprehensively grasp the various components of TCM, and establish a systematic and mature separation and purification strategy to ensure the scientificity and rigor of clinical dosing regimens.

(2) Screening for more effective immune components of TCM. Because TCM lacks the ability to kill tumor cells specifically and robustly, it is currently only used as an auxiliary therapeutic agent in anti-tumor treatment. Future studies should thus focus on discovering and screening out more effective and targeted anti-tumor immune components from TCM, in addition to screening out toxic by-products. The ultimate goal is to develop anti-tumor Chinese herbal medicines with independent intellectual property rights, accelerate the modernization of Chinese medicine and promote Chinese medicine globally.

(3) Integration of traditional Chinese and Western medicine. Despite its extensive history, TCM lacks a scientific and systematic knowledge base, with very few studies focusing on the efficacy, toxicity and side effects of TCM. Bridging this gap will require combining the advantages of TCM with the research methods of Western medicine. Utilizing advanced detection equipment, molecular-level analyses of drug molecules can be conducted to unravel the relationship between the bioactive compounds in TCM and tumor immunotherapy, providing key insights into their mechanisms and targets.

(4) Research data informatization. Current TCM research faces challenges such as small samples and short observation periods. Addressing these issues requires multi-disciplinary approaches, such as network pharmacology, large-sample multi-omics technology and big data mining. Collectively, these strategies could shed light on the molecular targets and signaling pathways through which TCM regulates immune function, thus contributing to the establishment of a comprehensive database to guide large-scale, high-quality clinical research plans.

## Figures and Tables

**Table 1:** Various modes of bioactive compounds of traditional Chinese medicine in the tumor microenvironment.

Compound	Model	Adjustment Mode	References
Saponins	HCC liver cancer (in vivo); Lewis lung cancer (in vivo)	TAMs	[23,24,25]
	H22 liver cancer (in vivo); Jurkat leukemia mice (in vivo); breast cancer (in vivo)	T-cells	[26,27,28,29]
	Lewis lung cancer cells (in vitro); cervical cancer Hela cells (in vitro)	PD-L1	[30,31]
Polysaccharides	BBN bladder cancer (in vivo); Lewis lung cancer (in vivo)	TAMs	[32,33,34,35]
	4T1 breast cancer (in vivo)	DCs	[36]
	B16F10 melanoma (in vivo); liver cancer (in vivo); CT26 colorectal cancer (in vivo)	T-cells	[37,38,39,40]
Flavonoids	CT26 colorectal cancer (in vivo);	DCs	[41]
	breast cancer cells (in vitro); Lewis lung cancer (in vivo); NSCLC mice (in vivo)	PD-L1	[42,43,44,45,46]
Alkaloids	Lung cancer patients (in vivo)	T-cells	[47]
	Lewis lung cancer (in vivo)	PD-L1	[48,49]

**Table 2:** Effects of bioactive compounds of traditional Chinese medicine on tumor-associated macrophages in the tumor microenvironment.

Form	Compound	Model	Mechanisms of Action	References
Saponins	Astragaloside IV	Huh-7 nude mice with hepatocellular carcinoma (in vivo)	Inhibition of M2 polarization through the TLR4/NF-κB/STAT3 signaling pathway inhibits tumor proliferation, invasion and migration	[23]
	Astragaloside IV	Lewis lung cancer mice (in vivo)	Blocks macrophage M2 polarization via the AMPK signaling pathway	[24]
	Ginsenoside	Lewis lung cancer mice (in vivo)	Reduces expression of the M2 macrophage markers CD206 and VEGF in vivo	[25]
Polysaccharides	Poria mushroom polysaccharide	BBN bladder cancer rats (in vivo)	Activates macrophages through the TLR4/NF-κB signaling pathway	[32,33,34]
	Astragalus polysaccharide PG2	Lewis lung cancer mice (in vivo)	Dose-dependently enhances M1 polarization and down-regulates IL-4-/IL-13-induced M2 polarization	[35]

**Table 3:** Effects of bioactive compounds of traditional Chinese medicine on dendritic cells in the tumor microenvironment.

Form	Compound	Model	Mechanisms of Action	References
Polysaccharides	Radix Astragalus Radix Conopsis	4T1 breast cancer mice (in vivo)	Stimulates DCs to express higher levels of CD80 and CD86; increases the infiltration of CD8+ T cell in tumors	[36]
Flavonoids	Gambogic acid	CT26 colorectal cancer mice (in vivo)	Stimulates maturation of DCs	[41]
Terpenes	Cryptotanshinone	Lewis lung cancer mice (in vivo)	Induces maturation of DCs in a MyD88-dependent manner	[62]
	Dihydrotanshinone	HCC liver cancer mice (in vivo)	Generates ROS effectively, enhances plumbagin-mediated ICD and promotes DC maturation	[64]
Quinones	Plumbagin	HCC liver cancer mice (in vivo)	Induces ICD to stimulate maturation of DCs	[64]

**Table 4:** Effects of bioactive compounds of traditional Chinese medicine on natural killer cells in the tumor microenvironment.

Form	Compound	Model	Mechanisms of Action	References
Anthraquinones	Emodin	A549 lung cancer cells (in vitro)	Enhances the killing effect of NKs on A549 by affecting the equilibrium state of signaling by NKs	[68]
Plant- extracted glycoproteins	ZPDC glycoprotein	Diethylnitrosamine (DEN)-induced hepatocellular carcinoma in mice (in vivo)	Secretes perforin and granzyme B and activates NKs	[69]
Terpenes	Lupeol	Gastric cancer HGC27 cells (in vitro)	Enhances the proliferative capacity and cytotoxicity of NKs against gastric cancer cells	[70]

**Table 5:** Effects of bioactive compounds of traditional Chinese medicine on T-cells in the tumor microenvironment.

Form	Compound	Model	Mechanisms of Action	References
Saponins	Ginsenosides	Human monocyte-derived DCs (in vitro)	Promotes the conversion of naive T cells to Th1 cells through DCs	[26]
	Ginsenoside Rg3	H22 liver cancer mice (in vivo)	Increases IFN-γ and IL-2 production	[27]
	Ginsenoside Rh2	Jurkat leukemia mice (in vivo)	May inhibit cell growth through the PI3K/Akt/mTOR pathway	[28]
	Saikosaponin A	Dimethyl-benthrathracene (DMBA)-induced breast cancer in rats (in vivo)	Transforms Th2 cells into Th1 cells	[29]
Polysaccharides	Ganoderma lucidum	B16F10 melanoma mice; HCC liver cancer mice	Induces the production of granzyme B and perforin to enhance the cytotoxicity of CTLS to melanoma cells; Inhibits Treg cells action by up-regulating miR-125b, leading to suppression of Notch1 signaling pathway and FoxP3 expression	[37,38]
	Licorice polysaccharide	H22 liver cancer mice; CT26 colorectal cancer mice	Activates CD4+ and CD8+ T cells and increases Th1/Th2	[39,40]
Alkaloids	Ligustrazine	Lung cancer patients (pre-clinical patients)	Reduces the expression of Th2 cytokines	[47]
Terpenoids	Elecampane	MC38 colorectal cancer mice	Increases the proportion of CD8+ T cells and M1 macrophages in the tumor microenvironment	[79]

**Table 6:** Modulation of PD-L1 by bioactive compounds of traditional Chinese medicine.

Form	Compound	Model	Mechanisms of Action	References
Saponins	Ginsenoside Rg3	Lewis lung cancer cells (in vitro)	Suppresses PD-L1 in LLC through the PI3K/Akt/mTOR pathway	[30]
	Astragaloside IV	Cervical cancer Hela cells (in vitro)	Inhibits PD-1 and PD-L1 expression through the P38 signaling pathway	[31]
Flavonoids	Hyperoside	MC38 colorectal cancer mice (in vivo)	Down-regulates PD-L1 and CD47 expression by degradation of c-Myc	[42]
	(-)-Sativan (SA)	Breast cancer cells (in vitro)	Inhibits PD-L1 expression by up-regulation of miR-200c	[43]
	Quercetin	Lewis lung cancer mice (in vivo)	Suppresses PD-L1 through the JAK2/STAT3 pathway	[44,45]
	Apigenin and lignans	NSCLC mice (in vivo)	Inhibits KRAS mutant lung cancer proliferation and down-regulates IFN-γ-induced PD-L1 expression	[46]
Alkaloids	Berberine	Lewis lung cancer mice (in vivo)	Specific binding to CSN5 leads to PD-L1 degradation	[48]
	Evodiamine	Lewis lung cancer mice (in vivo)	Increases CD8+ T-cell activity and down-regulates MUC1-C/PD-L1	[49]
Terpenoids	Paeoniflorin	HepG2 cells (in vitro)	Suppresses PD-L1 in HepG2 cells through the JAK/STAT3 pathway	[86]
	Celastrol	B16F10 melanoma mice (in vivo)	Down-regulates PD-L1 expression in tumor cells via the NF-κB pathway	[87]
	Andrographolide	Lewis lung cancer mice and NSCLC mice (in vivo)	Oxidatively inhibits STAT3 phosphorylation and p62 accumulation and regulates selective autophagic degradation of PD-L1	[88]

## Data Availability

Not applicable.
